# Supplementary material for: Integration of lymphatic vasculature to a human lymph node-on-chip enhances physiological immune properties
Source: Mater Today Bio. 2025 Sep 22;35:102326. doi: 10.1016/j.mtbio.2025.102326 (PMC12506468; doi:10.1016/j.mtbio.2025.102326)
Supplement: Multimedia component 1 [file mmc1.pdf]

Table S1. Human lymph node donor characteristics. F: Female, M: Male, DBD: Donation after Brain Death, DCD: Donation after Circulatory Death.

#	Donor	Sex	Age	HLA-type
1	DBD	F	59	A3 A28 A68 B12 B44 B70 B72 Bw4 Bw6 Cw2 Cw5 DR3 DR17 DR4 DR52 DR53 DQ2 DQ3 DQ7 DQA-03 DQA-05 DP-0401 DPA-01
2	DCD	F	46	A1 A11 B17 B57 B35 Bw4 Bw6 Cw4 Cw6 DR1 DR7 DQ1 DQ5 DQ3 DQ9 DQA-01 DQA-02 DP-0201 DPA-01
3	DCD	M	50	A1 A19 A33 B14 B65 B40 B61 Bw6 Cw2 Cw8 DR3 DR17 DR6 DR13 DR52 DQ1 DQ6 DQ2 DQA-01 DQA-05 DP-0201 DP-10 DPA-01 DPA-02
4	DBD	F	68	A2, A68 (28), Bw4, Bw6, B7, B53, Cw4, Cw7, DP-0401, DR52, DPA-01, DQ6 (1), DQ4, DQA-01, DQA-04, DR13 (6), DR8

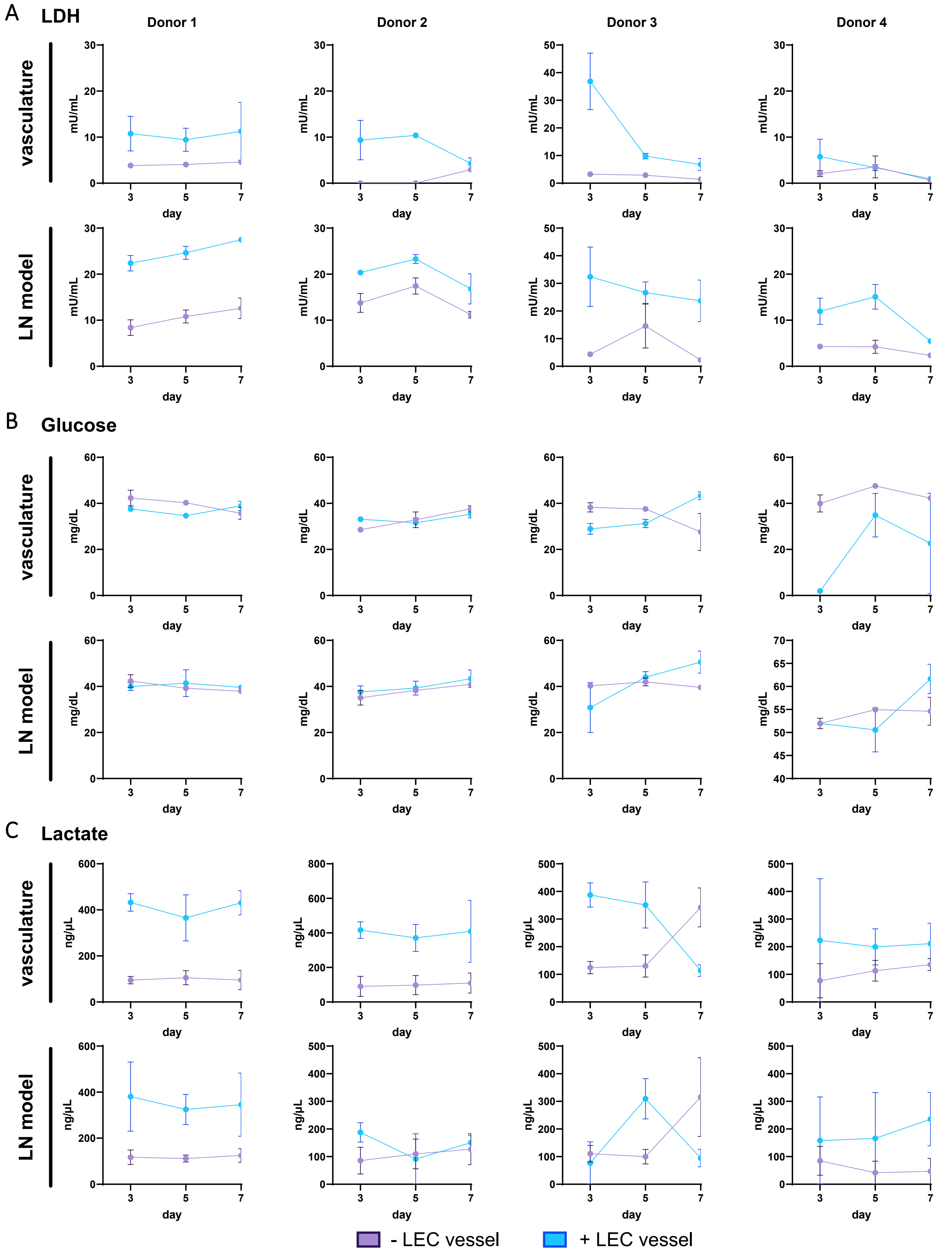

Fig. S1: Metabolic readouts of individual donors. A. LDH secretion **B.** Glucose concentration and **C.** Lactate secretion into culture supernatant of LN-on-chip cultures. Depicted are individual values for 4 different LN donors in duplicates.

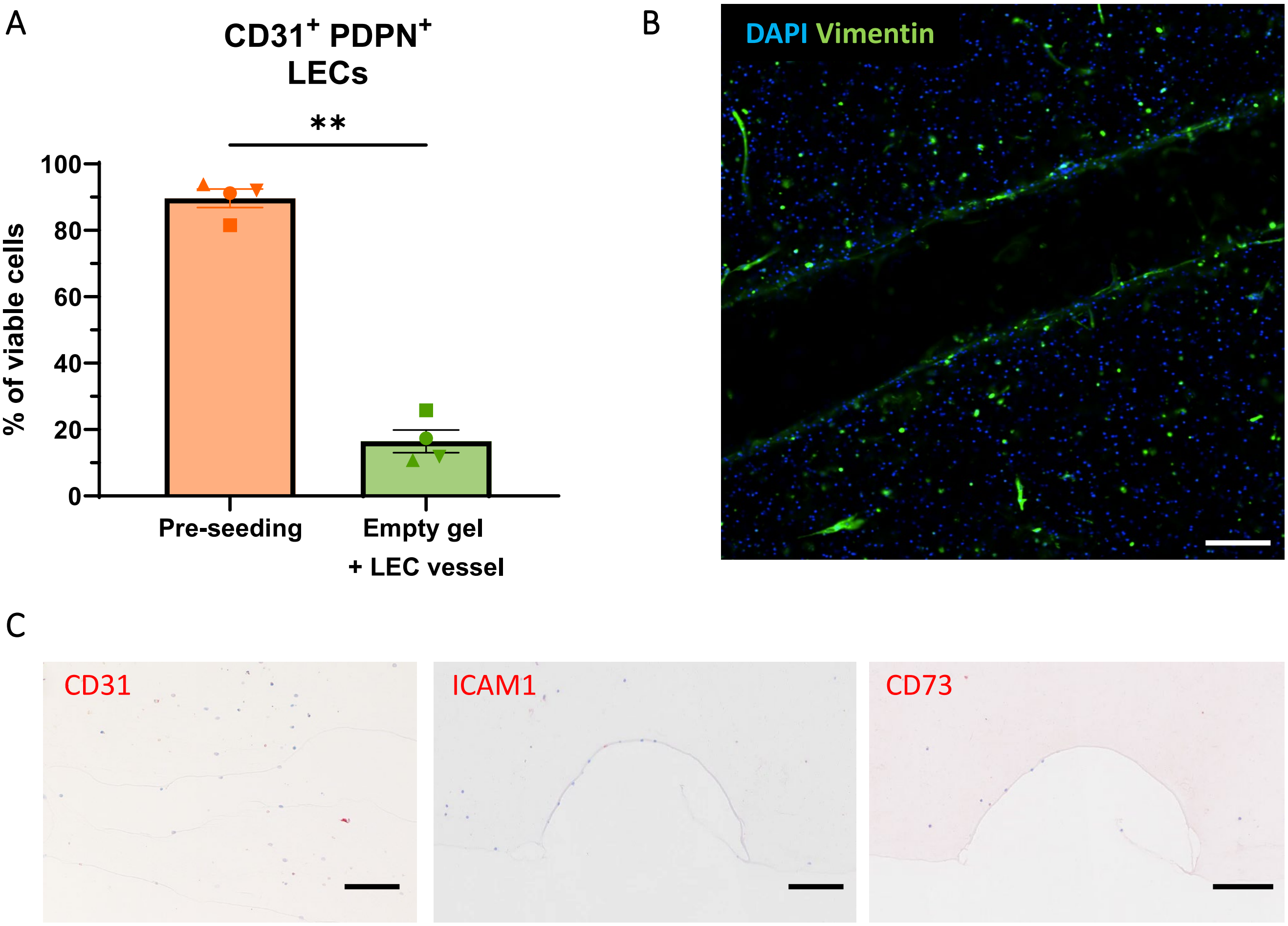

Fig. S2: LEC characterization. A. LEC phenotype from CD31 and PDPN marker expression of different donors used before seeding into the chip and after chip culture through an empty hydrogel **B.** Vimentin (green) and DAPI (blue) staining of a LN-on-chip with an empty vessel, imaged in the middle of the channel. Scale bar: 200 µm. **C.** Immunohistochemical staining of cross-sectional channel for LEC markers CD31, ICAM1 and CD73 in LN-on-chip without LEC vessel. Scale bars: 100 µm.

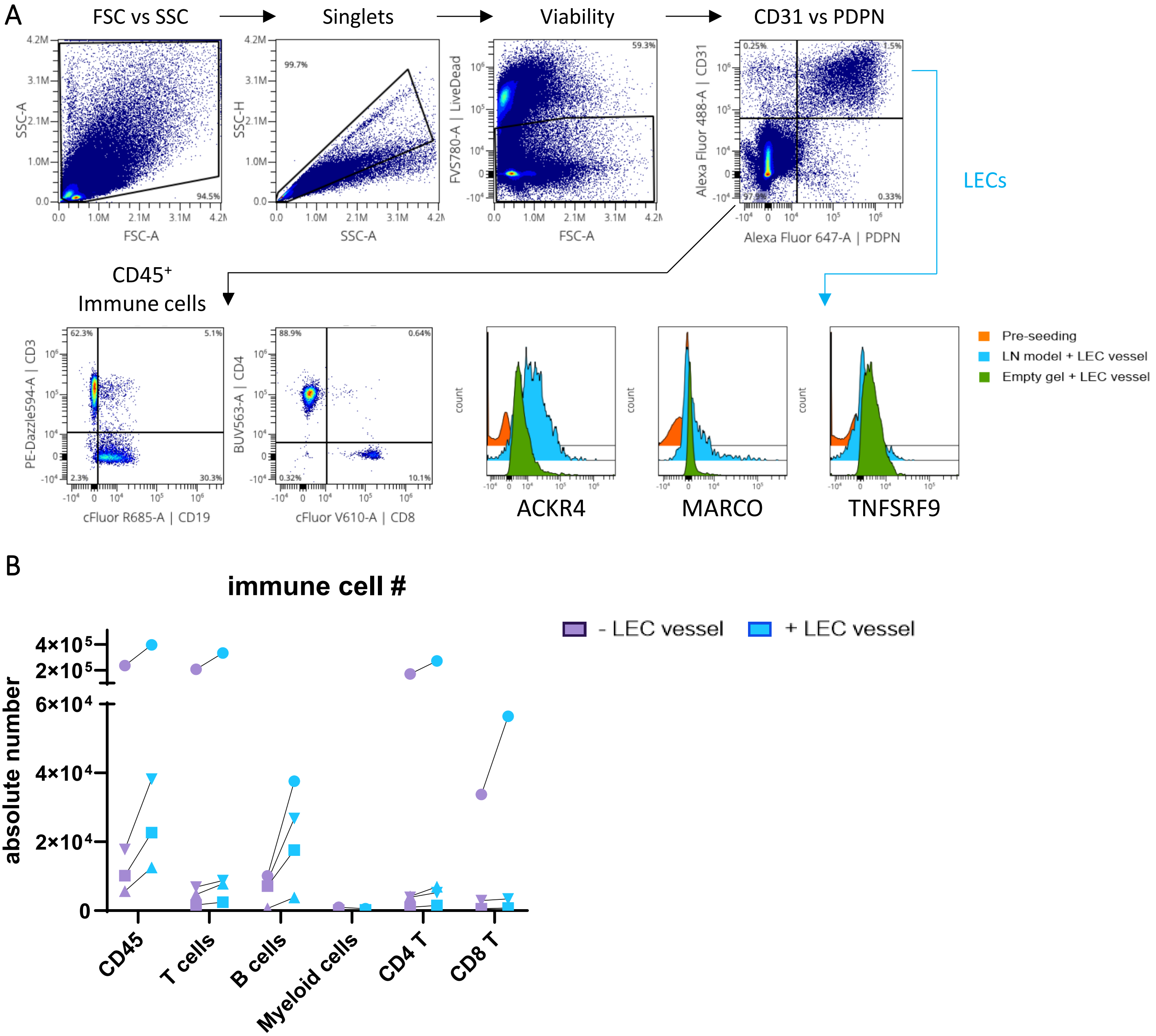

Fig. S3: Flow cytometry analysis. A. Representative gating strategy to identify different cells in LN-on-chip with LEC vessel, where $CD31^{+}PDPN^{+}$ LECs are selected for further phenotyping (blue line), and $CD31^{-}PDPN^{-}$ cells are selected for immune cell gating (black line). **B.** Absolute number of immune cell population in LN-on-chip ± LEC vessel. Shapes represent different donors; n = 4 independent experiments.

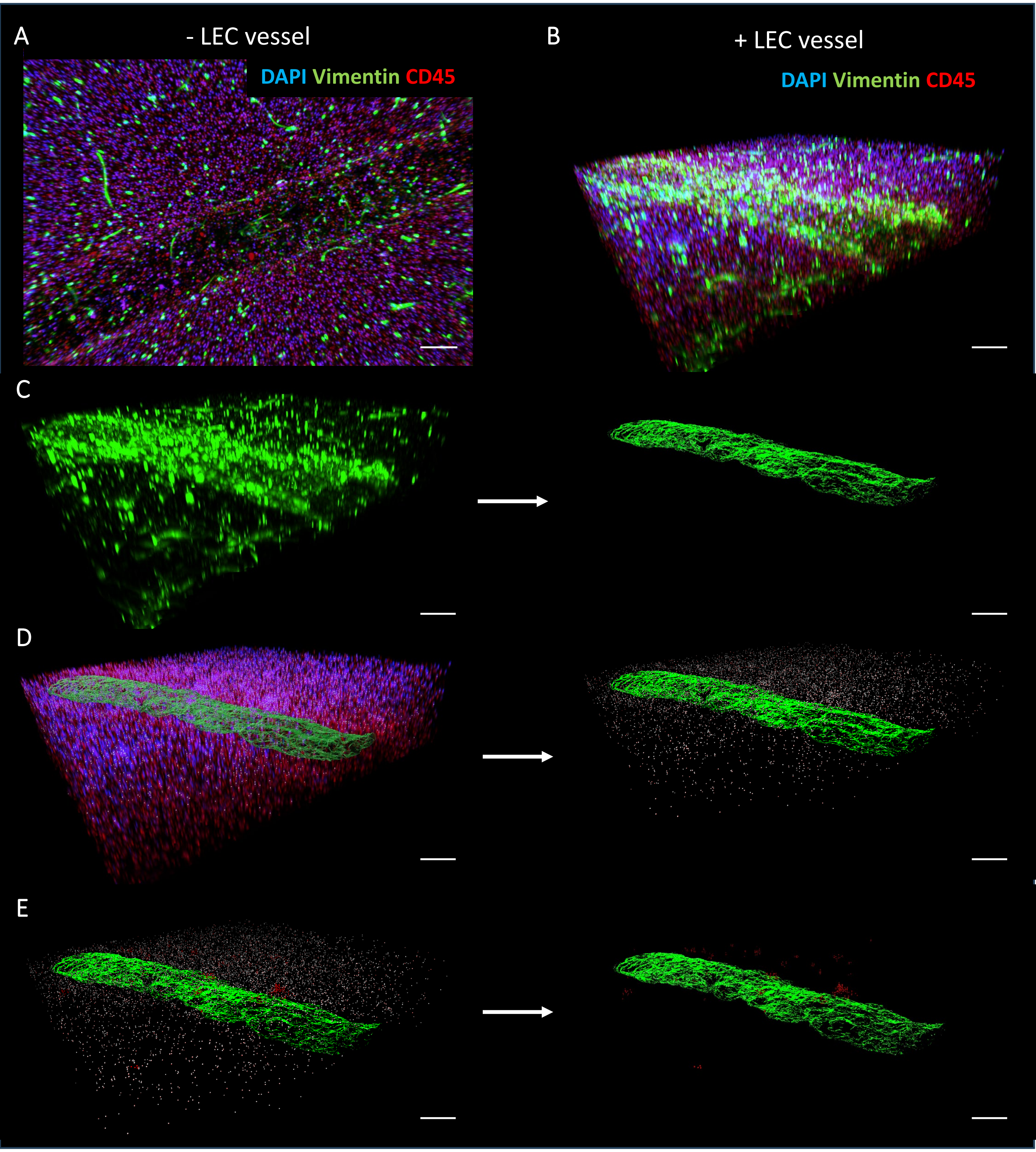

Fig. S4: LN-on-chip renderings at day 7. A. 3D projection of LN-on-chip without lymphatic vessel. **B.** 3D reconstruction of the LN-on-chip with lymphatic vessel stained with DAPI (blue), vimentin (green and CD45 (red)). **C.** Vessel surface rendering (green) based on vimentin. **D.** Immune cell rendering (grey) of DAPI^{+}CD45^{+} cells. **E.** Immune cells clusters rendered (red) based on the following criteria: 9 closest neighbours within an average distance of 0-20 µm. Scale bars: 200 µm.
